# Clinical characteristics and novel strategies of immune checkpoint inhibitor rechallenge therapy for non-small cell lung cancer: a comprehensive review

**DOI:** 10.3389/fimmu.2023.1309055

**Published:** 2024-01-12

**Authors:** Hao Zhang, Yujun Hu, Tingting Wu, Yeshan Chen, Bin Yang, Tao Xie

**Affiliations:** ^1^ Department of Radiation Oncology, Hubei Cancer Hospital, TongJi Medical College, Huazhong University of Science and Technology, Wuhan, Hubei, China; ^2^ State Key Laboratory of Oncology in South China, Guangdong Key Laboratory of Nasopharyngeal Carcinoma Diagnosis and Therapy, Guangdong Provincial Clinical Research Center for Cancer, Sun Yat-sen University Cancer Center, Guangzhou, China; ^3^ Department of Radiology, Sun Yat-Sen University Cancer Center, Guangzhou, China; ^4^ Department of Health Management, Hubei Cancer Hospital, TongJi Medical College, Huazhong University of Science and Technology, Wuhan, Hubei, China; ^5^ Institute of Radiation Oncology, Cancer Center, Union Hospital, Tongji Medical College, Huazhong University of Science and Technology, Wuhan, Hubei, China; ^6^ Department of Thoracic Oncology, Hubei Cancer Hospital, TongJi Medical College, Huazhong University of Science and Technology, Wuhan, Hubei, China

**Keywords:** immune checkpoint inhibitor, rechallenge, non-small-cell lung cancer, clinical strategies, review

## Abstract

Treatment of non-small-cell lung cancer (NSCLC) has entered the immunotherapy era, marked by significant survival improvements due to the use of immune checkpoint inhibitors (ICIs). However, owing to factors, such as disease progression, long-term use, and side effects, some patients discontinue immunotherapy, resulting in limited subsequent treatment option and a negative impact on their survival and quality of life. We have collected relevant data which reveal that ICI rechallenge may be an effective clinical strategy. However, many factors affect the efficacy of rechallenge, including patient characteristics, initial treatment drugs, treatment duration, efficacy, toxicity, and side effects. Additionally, the side effects of rechallenge and mechanisms of reversing drug resistance play crucial roles. Identifying suitable candidates, optimizing treatment plans and duration, enhancing treatment efficacy, and minimizing toxicity and adverse effects in rechallenges are pressing clinical needs. Addressing these issues can provide guidance for the clinical use of immunotherapy rechallenges to better serve patients. This review focuses on the clinical considerations and strategies for immune therapy rechallenges in NSCLC.

## 1 Introduction

Lung cancer, especially non-small cell lung cancer (NSCLC), ranks first in terms of incidence rate and mortality in China, posing a serious threat to human health (1). There have been some advances in surgery, radiotherapy, chemotherapy, and targeted therapy in treating this condition, and targeted therapy significantly improves the prognosis of patients with gene mutations; for example, compared with chemotherapy, postoperative adjuvant targeted therapy significantly improves the survival time and five-year disease-free survival rate of stage IIIA EGFR-positive NSCLC patients, with a 13.0% increase in five-year survival rate (2). However, despite these improvements, the 5-year survival rate of patients with advanced NSCLC remains not high (3). Targeted therapy can consider immunotherapy after drug resistance. Immunotherapy, with its high specificity and low side effects, has emerged as a promising approach in tumor treatment, significantly prolonging survival time, improving quality of life, and offering hope to those with advanced NSCLC (4).

Tumor immunobiology has opened up a new chapter for tumor immunotherapy. This change is based on the discovery of tumor immune checkpoints, the successful development of immune checkpoint inhibitors (ICIs), and advances in the generation of genetically modified immune cells. Compared with traditional anti-tumor therapy, immunotherapy has unparalleled advantages. It can utilize and mobilize the body’s immune system, enhance its ability to recognize tumor cells, block the inhibitory immune signals emitted by tumor cells, weaken the immunosuppressive ability of the tumor microenvironment (TME), and can benefit patients in the long term. This strategy brings new hope for cancer treatment and also provides more sustainable survival possibilities for cancer patients. However, there are still many issues that need to be addressed, such as the specific mechanisms of immunotherapy, biomarkers, drug resistance, and rechallenge after drug resistance (5).

During ICI treatment, some patients have to interrupt the therapy because of immune-related adverse reactions (irAEs) or disease progression (PD). The follow-up treatment for these patients is challenging, and sometimes physicians plan a new round of immunotherapy rechallenge. Although results show that this treatment plan brings some hope to patients, research data are limited. Immunotherapy rechallenge involves other treatment methods between two ICI regimens, and additional treatment can affect homeostasis of the patient’s immune system, leading to activation of the secondary immune response (6).

Research has shown that some patients with NSCLC experience recurrence after completing first-line treatment, with primary drug resistance occurring in a significant percentage, both in first-line (7.0–27.0%) and second-line (20.0–44.0%) treatments (7). Some of these patients respond positively to immunotherapy rechallenge; however, the specific mechanism is not yet clear (6). Immunotherapy rechallenge involves administering immunotherapy again after a previous course of treatment has been terminated for any reason. Based on existing research data, we believe that immunotherapy rechallenge in NSCLC is meaningful. This review explores the relevant aspects of immunotherapy rechallenge.

## 2 Rechallenge after the established course of immunotherapy

After completion of the established immunotherapy course, the immune response can be stimulated by immunotherapy rechallenge. This strategy of rechallenge is based on the short time of receiving immunotherapy, insufficient drug exposure, unstable receptor occupancy, and potential underutilization of the effectiveness of immunotherapy. There are relatively few observational studies on the efficacy of immunotherapy rechallenge in patients with NSCLC. However, some studies have reported rechallenge efficacy and safety, suggesting that this treatment has certain prospects. A study reported the immunotherapy rechallenge results of 17 NSCLC patients who had previously received treatment with ICI and experienced PD after discontinuation. Among them, 1 patient achieved partial remission (PR) and 9 patients achieved stable condition (SD) after receiving immunotherapy rechallenge (8).

Advancements in advanced NSCLC treatment have significantly extended survival (9). Immunotherapy is typically administered until disease progression or intolerable adverse effects persist for 2 years, which indicates that patients have achieved certain therapeutic effects after receiving immunotherapy. Research is currently focusing on exploring whether immunotherapy can be reconsidered once PD occurs after the completion of an established course. Keynote 010 and Keynote 024 trials showed disease control rates of 79.0% and 70.0% when pembrolizumab was reintroduced after initial treatment (10, 11). Checkmate 153 study found that 34 patients with advanced NSCLC who stopped taking nivolumab after receiving one year of treatment developed PD. After nivolumab rechallenge, the PD of the target and new lesions were 35.0% and 41.0%, respectively. The remaining patients achieved good control, suggesting that approximately 2/3 of patients could benefit from immunotherapy rechallenge (12). Durvalumab rechallenging after 1 year from the initial discontinuation resulted in 1-year progression-free survival (PFS) of 31.0% and a disease control rate of 52.4% in patients with PD (13). These clinical studies indicate that a high proportion of patients with PD after initial therapy with programmed death 1/programmed death-ligand 1 (PD-1/PD-L1), may still benefit from re-immunotherapy, achieving disease control.

It should be noted that these observational studies have some limitations, such as small sample size and patient selection bias. Therefore, large-scale studies are needed to evaluate the efficacy and safety of ICI rechallenge.

## 3 Further rechallenges after the progress of immunotherapy

Drug resistance is inevitable in the process of tumor drug treatment. This is because tumor cells have complex drug resistance mechanisms, namely 1) increased drug efflux: tumor cells expel drugs out of the cell by increasing the expression of proteins related to drug efflux, reducing the concentration of drugs inside the cell, and thus developing drug resistance; 2) target mutations or deletions: tumor cells may undergo genetic mutations or deletions, making the drug unable to bind to the target and thus lose its effectiveness; 3) activation of alternative pathways: tumor cells may activate other signaling pathways to replace pathways suppressed by drugs, leading to drug resistance; and 4) cell death resistance: tumor cells may resist drug-induced cell death by activating certain signaling pathways or expressing certain genes, leading to drug resistance (14). Currently, immunotherapy rechallenge after developing drug resistance has gained popularity. Initial trials in melanoma demonstrated that rechallenge can effectively regain disease control, with the same efficacy as initial immunotherapy and tolerable side effects (15, 16).

In clinical practice, if lung cancer patients do not harbor driver gene mutations, it is generally recommended to replace drugs with new immune ones or use a combination regimen to reverse resistance to immunotherapy when it develops (17). A total of 69 patients with metastatic renal cell carcinoma were included in a study. During the first ICI treatment (ICI-1), single ICIs (n = 27) or ICIs combined with targeted therapy (n = 29) were used. Most patients discontinued ICI-1 because of PD (n = 50) or toxic reactions (n =16). However, when switching to the second immunotherapy challenge (ICI-2), it was observed that some patients benefited, with the total effective rates of ICI-1 and ICI-2 being 37.0% and 23.0%, respectively, and it was found that among patients who had previously responded to ICI-1, ICI-2 was the drug most patients likely responded to [7/24 (29.2%)] (18).

Other studies also suggested that after resistance to the PD-1/PD-L1 regimen, the following combination regimens using ICIs can continue to provide clinical benefits (19): 1) immunotherapy combined with chemotherapy: 35 patients with advanced NSCLC who underwent immunotherapy were treated with pembrolizumab combined with chemotherapy. The results showed that PR was 23.5%, SD was 53.0%, and the percentage with treatment-related adverse reactions was 45.7%. There were no treatment-related deaths, indicating that the combination of pembrolizumab and second-line chemotherapy prolonged PFS in advanced NSCLC patients who advanced after immunotherapy (20). 2) Immunotherapy combined with antiangiogenic therapy: a study included 52 patients with unresectable liver cancer who received atezolizumab monotherapy for resistance. The combination of atezolizumab and bevacizumab therapy once again showed benefit. The study results showed that out of 26 patients, 1 patient achieved PR (objective response rate [ORR] 3.8%), 13 patients had SD, and the DCR was 53.8%. The ORR and DCR of patients who did not cross to Arm F1 after PD were 0.0% and 30.8%, respectively (21). 3) Among 29 patients with advanced NSCLC who developed after anti PD-1/L1 treatment, 2 patients achieved PR using pepinemab (SEMA4D inhibitor) combined with avelumab (PD-L1 inhibitor), with tumor reduction of 65.0% and 52.0%, respectively. One patient continued to benefit for more than 1 year and 5 patients ≥ 6 months, with a DCR of 58.6% (17/29) (22). 4) Bispecific antibodies: in a clinical phase II study, in patients with unresectable or metastatic melanoma who had failed single drug treatment with PD-1 inhibitors, pembrolizumab combined with low-dose aspirin monoclonal antibody was used for rechallenge. The results showed that the confirmed ORR was 29.0%, mPFS was 5.0 months, and mOS was 24.7 months. Moreover, a total of 29 patients with solid tumor who had failed previous ICI treatment were treated with KN046-302, and an ORR of 12.0% and mPFS of 2.69 months were achieved (23).

Currently, choosing between maintaining the initial plan or selecting new drugs for subsequent challenges lacks sufficient evidence. However, rechallenge reliably yields therapeutic effects, and its safety profile is acceptable.

## 4 Side effect of rechallenge post-immunotherapy

Immunotherapy, while clinically beneficial, can cause unique adverse effects known as irAEs, including potentially severe and fatal toxic reactions (24). Some patients experiencing significant irAEs during initial treatment with CTLA-4 and/or PD-1/PD-L1 inhibitors can safely receive immunotherapy again (25). The choice of treatment depends on multiple factors, including the severity and nature of the initial irAEs, the effectiveness of systemic immunosuppression, and the presence or absence of alternative treatment options. There is limited data on the benefits of reusing immunotherapy for patients experiencing irAEs for the first time, especially those achieving complete or sustained remission after initial treatment regimen without further intervention. Multidisciplinary discussions should be conducted to fully evaluate the pros and cons of patient re-therapy. Guo et al. conducted a retrospective analysis and found that 9.4% of patients with stage IV NSCLC discontinued PD-1 inhibitor treatment due to irAEs. When reusing immunotherapy, 60.0% of patients experienced recurrent or new irAEs, with nearly 50.0% being ≥ grade 2 irAEs, while no grade 4 irAEs or irAE-related deaths occurred. Timely treatment mitigates or eliminates these issues (26). In a meta-analysis, Zhao et al. found that the overall incidence of recurrent irAEs related to PD-1/PD-L1 inhibitor retreatment was 34.2%, with severe irAEs reaching 11.7% (27).

Previously, CTLA-4 inhibitors were used to manage toxicity, and PD-1/PD-L1 inhibitors were administered to prevent retreatment. In the CheckMate 172 study, progress was made by implementing a regimen involving aspirin followed by nivolumab, with the longest duration being 2 years. Among the 84 patients who initially experienced grade 3 or higher irAEs related to aspirin, no recurrence of grade 3 or higher diarrhea or colitis occurred (28). In another group of 67 patients with advanced melanoma treated with either pembrolizumab or nivolumab, serious irAEs emerged. Consequently, 34.0% of these patients experienced new irAEs, whereas only 3.0% of the patients experienced recurring irAEs associated with ipilimumab (29).

Further, 80 patients with advanced melanoma were treated with PD-1/PD-L1 inhibitors following CTLA-4/PD-1 inhibitor-related toxicity. These patients initially received CTLA-4 and PD-1 inhibitors, leading to initial irAEs, and were subsequently treated with either nivolumab or pembrolizumab monotherapy. Among them, 18% experienced recurrent irAEs, including one fatality due to Stevens–Johnson syndrome. Other recurring grade 3 irAEs included hepatitis (7.0%), hepatitis (3.0%), dermatitis (3.0%), elevated lipase levels (3.0%), and pituitary inflammation (1.0%) (30).

In another study including 167 patients with malignant tumors who received immunotherapy, 32 were rechallenged with CTLA-4 inhibitors, while 135 were rechallenged with PD-1/PD-L1. Of those, 44.0% experienced recurrence when treated with anti-CTLA-4 inhibitors, and 32.0% experienced recurrence when treated with anti-PD-1/PD-L1 (31).

After experiencing toxicity from prior PD-1/PD-L1 inhibitor treatment, a combination of anti-PD-1/PD-L1+CTLA-4 inhibitors were given for blocking the irAEs and retreatment. In a phase II clinical study involving the use of durvalumab combined with tremelimumab to treat patients previously treated with PD-1/PD-L1 inhibitors, 58 patients were enrolled. Among them, 28 were initially resistant to the drug, and 30 had developed resistance over time. This study revealed that the objective response rate (ORR) in the initially drug resistant group was only 7.0%, while it was 0.0% in the acquired resistance group. PFS for both groups was only 2 months, and OS was 7.6 months. Despite the limited efficacy of the dual-immunotherapy combination of durvalumab and tremelimumab in the population previously treated with immunotherapy, initial results have shown promise, providing new strategies for addressing refractory tumors (32).

## 5 Biomarkers of immunotherapy rechallenge

At present, immunotherapy rechallenge can only benefit a small number of patients, and how to identify this population has attracted increasing attention. Identifying the characteristics of potential beneficiaries of the rechallenge strategy is crucial. This will allow the screening of groups with good clinical response to immunotherapy and prediction of treatment outcomes. Commonly used immune efficacy markers include PD-1/PD-L1 expression and tumor mutational burden (TMB) (33, 34). Among them, the expression level of PD-L1 is the main biomarker. However, physicians also recognize its limitations. At present, no conclusion has been drawn to determine which patients can benefit from immunotherapy rechallenge, and we believe that this is a hot topic for future research.

According to clinical studies, including KEYNOTE-010, KEYNOTE-024, and CheckMate-026, PD-L1 expression levels and TMB are the most commonly used biomarkers (35). Unfortunately, retesting PD-L1 before rechallenge is rarely performed in the real world (36). A retrospective study examined 11 NSCLC patients who underwent nivolumab/pembrolizumab rechallenge after suspending initial nivolumab treatment. It was found that 5 patients exhibiting reactions (PR and SD) had high PD-L1 expression (37). However, another study analyzing 35 NSCLC patients who received immunotherapy rechallenge from six Japanese institutions did not find a correlation between the efficacy of rechallenge and PD-L1 expression levels (38). Therefore, it is currently unclear whether the expression level of PD-L1 can reliably predict the outcome of immunotherapy rechallenge. In many retrospective studies, the expression level of PD-L1 was only determined during the initial ICI treatment, and we do not know whether the expression level of PD-L1 changed after the initial immunotherapy. We believe that it is important to measure it before challenging again.

The response and toxic side effects of immunotherapy are largely influenced by the TME. Therefore, researchers have explored the relationship between inflammatory indicators and immune therapy responses and toxic side effects, such as the ratio of neutrophils to lymphocytes (NLR), lymphocyte to monocyte ratio (LMR), and platelet to lymphocyte ratio (PLR), to predict immunotherapy outcomes (39, 40). Some inflammatory indicators can be used as a reference for the rechallenge effect of ICIs. Yuki et al. found that baseline blood NLR, LMR, and PLR values may be useful tools for predicting immunotherapy rechallenge treatment responders, consistent with observations at initial ICI treatment. Therefore, when considering immunotherapy rechallenge for NSCLC patients, inflammatory markers, such as NLR, LMR, and PLR, may help identify ICI re-responders to a certain extent (41). However, the mechanism is still unclear and requires further research.

## 6 Characteristics of adverse reactions of immunotherapy rechallenge

Although there is currently limited research on immunotherapy rechallenge, with the continuous development of immunology and biomedical science, our understanding of immunotherapy rechallenge is gradually increasing. To better understand the side effects of immunotherapy rechallenge, researchers are working to explore the specific mechanisms of immunotherapy rechallenge-related side effects, including research on cell signaling as well as immune cell activation and regulation. By delving deeper into these mechanisms, they hope to find ways to regulate immunotherapy rechallenge to reduce or avoid the occurrence of side effects. In addition, researchers are conducting clinical trials and observational studies to evaluate the safety and effectiveness of immunotherapy rechallenge. They are closely monitoring the adverse reactions that occur during immunotherapy rechallenge and attempting to find methods to predict and respond to these reactions. By analyzing and summarizing clinical trial data, they can continuously improve the strategies for immunotherapy rechallenge, improve the success rate of treatment, and enhance patient safety.

Predicting the safety of immunotherapy rechallenge involves assessing risk factors related to irAEs. Currently, there is no consensus on whether there is a potential connection between irAEs that occur during the initial immunotherapy process and the occurrence of recurrent/new irAEs during rechallenge. However, research has shown that when the initial irAEs are more severe, last longer, and require glucocorticoid or immunosuppressive therapy, the recurrence or incidence rates of irAEs tend to be higher during rechallenge (42). Some studies have indicated that immunotherapy rechallenge can be effective, with approximately 25.0–30.0% of patients experiencing irAEs similar to those observed during their initial treatment (43). If future data can further support specific characteristics of initial irAEs that predict the risk or severity of irAEs during rechallenges, it would provide valuable guidance for clinical decision making by physicians.

Additionally, the toxicity spectrum and severity of irAEs induced by anti-CTLA-4 and PD-1/PD-L1 antibodies differ (44). Research has shown that the incidence of irAE is higher in patients using anti-CTLA-4 (ipilimumab) than in patients using anti-PD-1/PD-L1 (45). The highest incidence rate and high level of irAEs are usually associated with the combination treatment of ipilimumab plus anti-PD-1/PD-L1 (46). In a large meta-analysis of 16485 patients, ipilimumab was more commonly associated with colitis and hypophysitis, while anti PD-1/PD-L1 was more commonly associated with diabetes and pneumonia (45). Whether these differences affect the safety of the rechallenge when selecting the initial and rechallenge drug types and sequences during immunotherapy is also a highly concerning issue in clinical practice.

In summary, although the side effects of immunotherapy rechallenge are a complex and challenging issue, researchers are working hard to better understand and control the adverse reactions during the immunotherapy rechallenge process. With the continuous progress of science and deepening of research, it is believed that there will be more effective methods in the future to manage the side effects of immunotherapy rechallenge, thereby improving its efficacy and safety.

## 7 The mechanisms of resistance to immunotherapy

Immunotherapy has completely changed the treatment of lung cancer in the past decade. By reactivating the body’s immune system, the survival period of some patients with advanced NSCLC has been significantly prolonged. However, resistance to immunotherapy is common, manifested as a lack of initial response or clinical benefits (primary resistance) or tumor progression after the initial response period (acquired resistance). Owing to the complex and dynamic interactions between malignant tumor cells and defense systems, overcoming the challenge of immunotherapy resistance is challenging (47).

**Figure 1:**
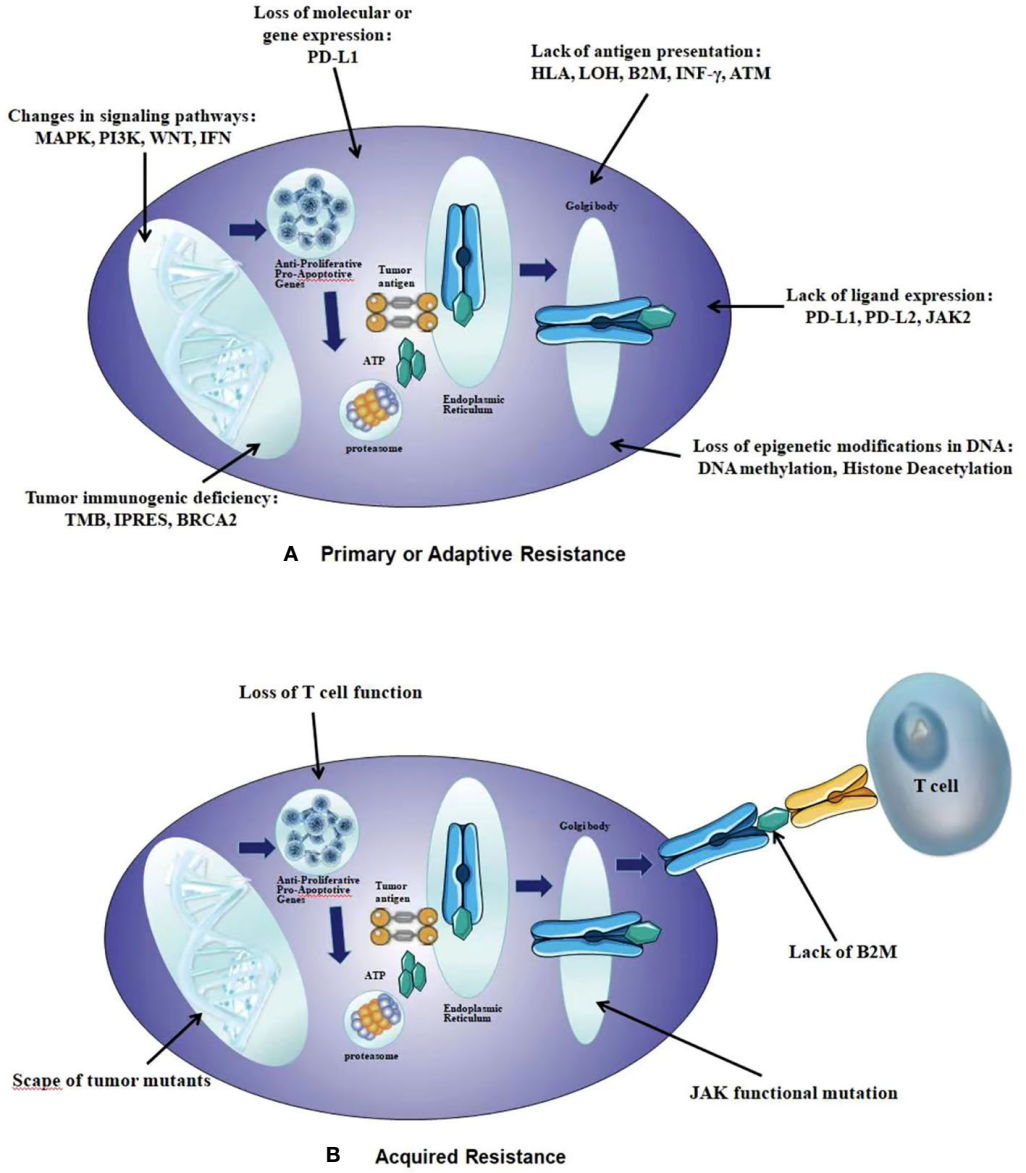
The intrinsic mechanisms of resistance to immunotherapy. **(A)** Primary or Adaptive Resistance: the expression or inhibition of certain genes and pathways, tumor cells inhibit anti-tumor T cell responses, tumor cell mutations, Tregs cells inhibit effector T cells; MDSCs cells inhibit immune function, M2 macrophages inhibit T cells, IFN γ promoting the expression of immunosuppressive molecules. **(B)** Acquired Resistance: anti-tumor T cells alter their functional phenotype and cease to exert their cytotoxic activity, genetic defects in B2M, MHC molecules act on T cells, the expression of some inhibitory immune checkpoint molecules in the tumor microenvironment.

**Figure 2:**
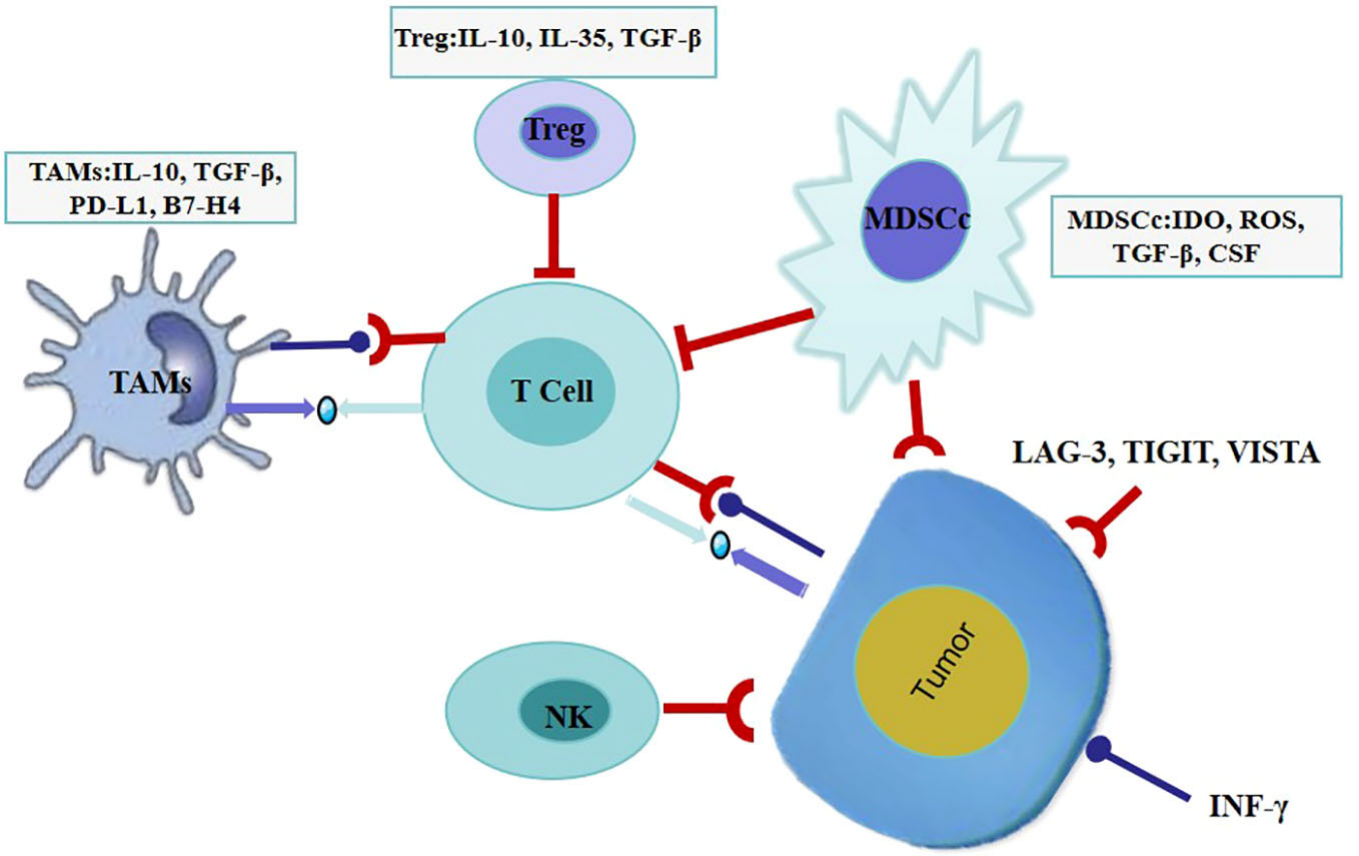
Tumor extrinsic mechanisms of resistance to immunotherapy.

The basic mechanisms of primary drug resistance widely vary, including tumor factors: the expression or inhibition of certain genes and pathways in tumor cells, which prevent immune cells from infiltrating or playing a role in the tumor microenvironment; Tumor cells may inhibit anti-tumor T cell responses; Tumor cell mutations can prevent interference with interferon-γ (IFN-γ). The signal response provides an advantage for tumor cells to escape from T cells, leading to primary resistance against PD-L1/PD-1 or CTLA-4 therapy. and host factors: Tregs cells directly or indirectly inhibit effector T cells; MDSCs cells directly inhibit immune function; M2 macrophages inhibit T cells through PD-L1/B7-H4 on the surface of tumor cells; IFN-γ promoting the expression of immunosuppressive molecules to inhibit the function of effector T cells, leading to immunosuppression and immune resistance. The possible mechanisms of acquired resistance overlap at least partially with those of primary resistance, mainly including: anti-tumor T cells alter their functional phenotype and cease to exert their cytotoxic activity; Genetic defects in B2M will lead to a lack of recognition by effector T cells; MHC molecules act on T cells, causing them to undergo mutations, deletions, mutations, or epigenetic changes in the new antigenic epitope; The expression of some inhibitory immune checkpoint molecules in the tumor microenvironment may lead to acquired resistance to checkpoint blockade therapy (**Figures 1**, **2**) (48).

The progress of immunotherapy can be divided into oligoprogression and systemic progression. Oligoprogression refers to the stabilization/remission of the primary tumor, with only 1–2 new metastatic foci present, and good tolerance to immunotherapy. Further immunotherapy combined with local treatment may be considered. Oligoprogression is more common in ICI resistant populations, and the prognosis is better than that of systemic progression. Systemic progression refers to the progression of tumors throughout the body after PD-1/L1 monoclonal antibody treatment for systemic drug resistance (49). In this case, it is usually necessary to consider changing the treatment plan. Researchers have explored therapies for ADC drugs, anti-angiogenic drugs, other ICIs, targeted therapy, antigen vaccines, cell therapy, low-dose radiotherapy, and gut microbiota improvement. For ADC and anti-angiogenic therapy, current phase II/III research data show great potential for application, while other ICIs (such as those targeting TIGIT, LAG-3, and TIM-3 checkpoints) and low-dose radiation therapy still need more research data to test their efficacy in immuno-resistant populations (50).

## 8 Strategies for reversing resistance to immunotherapy

**Figure 3:**
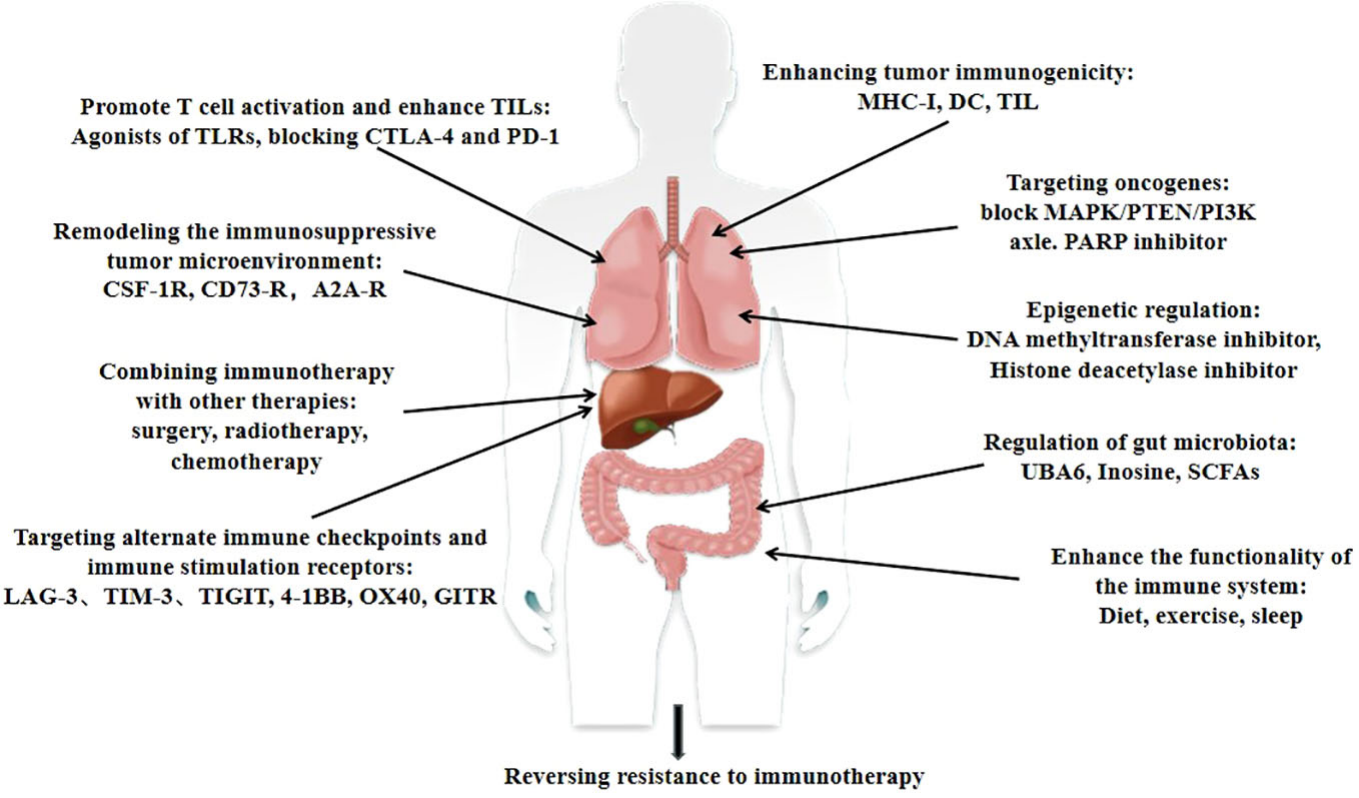
Strategies for reversing resistance to immunotherapy.

The methods and molecular mechanisms for reversing immune resistance are a complex and diverse field, and the specific mechanisms may vary depending on tumor type, individual differences, and the severity of drug resistance. However, some common strategies and molecular mechanisms can be summarized as follows (**Figure 3**):

1) Enhancing tumor immunogenicity: By increasing the antigen expression of tumor cells, the ability of tumor cells to be recognized by the immune system is enhanced, thereby enhancing the immunogenicity of tumor cells. This can be achieved through gene transduction, chemicals, or other methods; regulation of the composition and function of immune cells, such as CTL, MDSCs, and Tregs in the TME, as well as molecules expressed on tumor cells; upregulation of MHC-I expression to enhance tumor antigen presentation and anti-tumor immune response through chemically induced tumor cell apoptosis, thereby restoring the immune system’s recognition of tumors; and induction of tumor cell apoptosis to increase antigen exposure, enhance inflammatory response, increase DC activation, upregulate pro-inflammatory cytokines, leading to an increase in TIL, and promote cancer recurrence through non-redundant immune mechanisms (51).
2) Targeting oncogenes: Through gene therapy or other methods, targeting oncogenes inhibits the abnormal growth and proliferation of tumor cells, thereby reducing the immune evasion ability of tumor cells. Blocking the MAPK/PTEN/PI3K axis, using BRAF, MEK, and PI3K inhibitors, helps with Teff amplification, avoids T cell depletion and apoptosis, activates immune stimulation transcription programs, and promotes the production of pro-inflammatory cytokines and T cell toxicity. PARP inhibitors, as co-activators of CD8+T cell-mediated anti-tumor responses, although upregulated, can be complementarily suppressed by anti-PD-L1 therapy (52).
3) Promoting T cell activation and enhancing TILs: Vaccines, cytokines, or other methods promote T cell activation and expansion, increase T cell infiltration and activation in tumor tissue, and thus enhance T cell-killing effect on tumor cells. For example, blocking CTLA-4 and PD-1 enhances T cell initiation, Treg depletion, and CTL-mediated immune response through more antigen recognition, while PD-1 inhibitors participate in the later reactivation of the Teff response (53).
4) Remodeling the immunosuppressive TME: By regulating cytokines, chemokines, and other molecules in the TME, the immunosuppressive state of the TME is altered, and the activation and function of immune cells are improved. For example, double blockade of CTLA-4 and PD-1 promotes Treg cell penetration through tumors and reverses the immunosuppressive TME (54).
5) Targeting alternate immune checkpoints and immune stimulation receptors: Targeting backup immune checkpoints and immune stimulation receptors further enhances the function of the immune system and its killing ability against tumor cells. Moreover, blocking alternate immune checkpoints, such as LAG-3, and immune stimulation receptors, such as 4-1BB, OX40, and GITR, enhances effector T cell dilation and function, while controlling Treg cell inhibition function (55, 56).
6) Epigenetic regulation: Regulating epigenetic modifications affects gene expression and transcription, thereby altering the phenotype and function of tumor cells as well as enhancing immunogenicity and sensitivity to immunotherapy. For example, it makes tumors sensitive to PD-L1 blockade and increases the secretion of the immune-stimulating chemokines CXCL10 and CXCL9 to enhance immunogenicity and antigen presentation (57).
7) Regulation of gut microbiota: Regulating the composition and function of the gut microbiota affects the host’s immune system and metabolism, thereby affecting the occurrence and development of tumors and improving the response to immunotherapy (58).
8) Combining immunotherapy with other therapies (such as surgery, radiotherapy, and chemotherapy) to maximize treatment effectiveness and patient survival (59).
9) Enhancing the functionality of the immune system: This enhances the immune system’s ability to recognize and kill tumor cells, thereby improving the effectiveness of immunotherapy (60).

## 9 Discussion

Many patients will benefit from the continuous emergence of new ICIs. The potential of ICIs in the field of cancer treatment has attracted increasing attention, and researchers are constantly exploring new treatment strategies to achieve better therapeutic effects. This article evaluates the immunotherapy rechallenge and recognizes that patients can benefit from this treatment approach. Many factors can increase the complexity of treatment and affect the outcome of immunotherapy rechallenge, such as the clinical and pathological characteristics of patients, different rechallenge strategies, the duration of treatment interruption, and other treatments before rechallenge. However, most confirmed clinical studies are based on retrospective analysis of other immunotherapy clinical studies, and in addition, some studies had a small sample size, resulting in conflicting results in different studies (61).

Currently, most rechallenge studies are small-scale retrospective analyses, limiting the quality of evidence. Large-scale prospective cohort studies are needed in the future to investigate the feasibility and safety of rechallenges in patients with advanced NSCLC (62). Immunotherapy rechallenge is considered an alternative for these patients. While most rechallenge-related irAEs are mild and easy manageable, the potential for rare, fatal events necessitates continuous safety management. Furthermore, establishing a multidisciplinary, long-term, timely, complete, and closely connected monitoring model is essential.

To enhance treatment effectiveness, future research should focus on accurately identifying the characteristics of potential rechallenge beneficiaries, refining admission criteria and improving treatment efficiency. Currently, owing to their inherent limitations, most studies on rechallenge have low levels of evidence. Large-scale prospective cohort studies are needed to explore the feasibility and safety of rechallenges.

Based on effective survival benefits, immunotherapy rechallenge is a promising way to unleash the underutilized potential of immunotherapy, but there are also certain issues. Future research needs to address the following issues: how to identify patients who are most likely to benefit from immunotherapy rechallenge; how to choose the best rechallenge treatment plan for the target population; and how to maximize the therapeutic efficacy and safety of immunotherapy rechallenge while minimizing adverse reactions. Addressing these issues will help establish standardized treatment plans and apply them to routine clinical practice.

## Author contributions

TX: Data curation, Formal analysis, Supervision, Writing – review & editing, Funding acquisition. HZ: Writing – original draft. YJH: Data curation, Supervision, Writing – original draft. TTW: Data curation, Investigation, Writing – original draft. YSC: Data curation, Formal analysis, Resources, Supervision, Writing – review & editing. BY: Funding acquisition, Writing – original draft.
